# Receptor Binding by Cholera Toxin B-Subunit and Amino Acid Modification Improves Minimal Peptide Immunogenicity

**DOI:** 10.5402/2012/170676

**Published:** 2012-07-15

**Authors:** Andreas Boberg, Alexandra Stålnacke, Andreas Bråve, Jorma Hinkula, Britta Wahren, Nils Carlin

**Affiliations:** ^1^Department of Microbiology, Tumor and Cell Biology, Karolinska Institute, 171 82 Stockholm, Sweden; ^2^Division of Education and Research administration, Mälardalen University, P.O. Box 883, 721 23 Västerås, Sweden; ^3^Etvax AB, Gunnar Asplunds Alle 16, 171 63 Solna, Sweden; ^4^Institution of Clinical and Experimental Medicine, Linköping University, 581 83 Linköping, Sweden

## Abstract

We increase our understanding of augmenting a cellular immune response, by using an HIV-1 protease-derived epitope (PR_75–84_), and variants thereof, coupled to the C-terminal, of the B subunit of cholera toxin (CTB). Fusion proteins were used for immunizations of HLA-A0201 transgenic C57BL/6 mice. We observed different capacities to elicit a cellular immune response by peptides with additions of five to ten amino acids to the PR epitope. There was a positive correlation between the magnitude of the elicited cellular immune response and the capacity of the fusion protein to bind GM-1. This binding capacity is affected by its ability to form natural pentamers of CTB. Our results suggest that functional CTB pentamers containing a foreign amino acid-modified epitope is a novel way to overcome the limited cellular immunogenicity of minimal peptide antigens. This way of using a functional assay as readout for improved cellular immunogenicity might become highly valuable for difficult immunogens such as short peptides (epitopes).

## 1. Introduction

Combinations of antiretroviral drugs are still the only effective approach to delay the progression to acquired immuno-deficiency syndrome (AIDS) in HIV-1 infected patients. Due to the ability of the virus to introduce and tolerate mutations within the viral proteins, resistance to antiretroviral drug treatment may occur [1–3]. Approximately 10% of the newly diagnosed HIV positive patients, naïve to drug treatment in Europe, are infected with drug-resistant virus [4, 5]. Effective antiretroviral drug regimens are available; however, to a lesser extent in resource-poor countries than in the industrial parts of the world, whereby a vaccine strategy working in synergy with drug treatment would be beneficial. Previous work has shown that it is possible to induce a cellular immune response against mutated HIV-related epitopes by immunizing with such an epitope [6–8]. The use of short peptides as vaccine candidates is an interesting approach; however, there are immunogenicity problems using only the naked peptide as an immunogen. In order to increase the immunogenicity of short epitopes, novel approaches are needed. We have previously tested the approach of linking peptide to erythrocytes and using these cells as a vessel for transport of peptides to immune cells, as the erythrocytes were treated to be recognized as old by the cells [6]. Despite an increased response, the overall magnitude of the response was weak. We have also previously investigated the use of the B subunit of Cholera toxin (CTB) from the bacterium *Vibrio cholerae* as a carrier of an HIV-derived epitopes [9]. In the earlier work, we focused to target the immune response to HIV reverse transcriptase since that protein is heavily mutated during the viral life cycle, especially under suboptimal drug treatment. In the present study, an approach using modulated CTB as carrier of HIV derived protease peptides was initiated. The focus was set to HIV protease-(PR) derived epitopes already containing amino acid changes related to drug resistance. CTB has been shown to enable the induction of an antibody response to epitopes carried by full proteins [10–12], but less is known of its ability to augment cellular immune responses to short epitopes [9]. In that earlier work, pentamers of rCTB spontaneously formed. Shifting the setting to target short peptides as a load to the CTB molecule, it was hypothesized that we might augment immunoreactivity to the short peptide. 

Interestingly, there seemed to be a good correlation between a theoretical alpha amphipathic region in the C-terminal region and the ability of the constructs to form pentamers.

## 2. Material and Methods

### 2.1. Epitope Selection

An epitope from the HIV protease protein corresponding to amino acids 75–84 was selected for genetic conjugation to the B-subunit of the cholera toxin. The reason for this selection is that in an HIV infected individual it may become possible to immunize against development of drug resistance [7, 8]. The epitope was altered to include mutations, either the I84V mutation alone or the V82F and I84V mutations simultaneously. These epitopes are restricted to human leukocyte antigen (HLA) A0201 binding [7, 13], and naturally immunogenic in HIV-1 infected patients [13–15].

### 2.2. Production of Fusion Protein

Forward and reverse oligonucleotides corresponding to amino acid sequences 75–84, 75–89, and 75–94 from HIV protease harboring the mutations I84V and/or V82F were purchased from SGS DNA, Köping, Sweden. The oligonucleotides were made to encode overhangs corresponding to HindIII and Kpn1 restriction enzyme sites, to enable cloning into an expression vector [16]. This vector encodes the CTB under the control of a *tac* promoter [12, 16]. DNA-sequencing, and control cleavage using HpaI or BanII confirmed correct DNA sequences of the constructs. The constructs were electroporated into BL21 bacteria (Invitrogen) and expression of the fusion proteins was induced by isopropyl *β*-D-1-thiogalactopyranoside (IPTG; Sigma Aldrich, Stockholm, Sweden) stimulation for 3 hours at a concentration of 1mM. Fusion proteins were released from inclusion bodies using Bugbuster lysis (Novagen, Nottingham, United Kingdom) or by disruption of the BL21 cells using the constant cell disruption system (Constant Systems Limited) at 1.7 kBar.

### 2.3. Fusion Protein Characterization

The fusion proteins, hereafter called rCTB-PR_75–94_I84V, rCTB-PR_75–94_V82F/I84V, rCTB-PR_75–89_I84V, and rCTB-PR_75–84_I84V, were purified with either affinity chromatography and/or by using an ion-exchange column followed by affinity chromatography. The integrity of the protein was analyzed by N- and C-terminal sequencing, as well as by electrospray-mass-spectroscopy at the Protein Analysis Centre, Karolinska Institute, Sweden. Gel electrophoresis followed by silver staining verified the purity and configuration of the proteins. Biologic activities (GM-1 binding) of the CTB moiety of the fusion proteins were analyzed using the Biacore system. Theoretical analysis of the secondary structure of the fusion protein was done using the DNAStar Lasergene 8.1 program Protean (Bioinformatics Pioneer DNAStar, Inc., WI). The software predicts the secondary structure characteristics based on relevant method; in this case, the Eisenberg method [17] selected.

### 2.4. Immunizations

Groups of HLA-A0201 transgenic C57BL/6 mice [18], 3–8 mice per group, were housed and handled according to animal care guidelines at the Karolinska Institute, Sweden, with the approval of the local ethical animal welfare committee. Four immunizations were given subcutaneously at the base of the tail at a monthly interval. A total dose of 50 *μ*g immunogen in PBS was given in each immunization. The animals received one of the fusion proteins rCTB-PR_75–94_I84V, rCTB-PR_75–94_V82F/I84V, rCTB-PR_75–89_I84V, or rCTB-PR_75–84_I84V. Additional groups of mice were immunized with the mixture of rCTB and one of the two peptides PR_75–84_I84V or PR_75–84_V82F/I84V, rCTB only, the individual peptides PR_75–84_I84V or PR_75–84_V82F/I84V only, or were left untreated.

### 2.5. Serum Collection and Cell Preparations

Two weeks following each of the immunizations, individual blood samples were taken. Sera were individually collected and stored at −20°C until use. The peripheral blood mononuclear cells (PBMCs) from group-wise-pooled blood were separated by Ficoll-Paque gradient (GE Healthcare, Uppsala, Sweden). After washing, the PBMCs were resuspended in 1 mL RPMI medium containing 1% PEST, 2 mM L-glutamine, 1% HEPES, and 20% fetal calf serum (referred to as RPMI plus medium).

At the end of the studies, animals were sacrificed by cervical dislocation, and the spleens and serum harvested. Spleens were individually mashed in PBS, and the cell suspensions were separated by Ficoll-Paque gradient and resuspended in 2 mL RMPI plus medium. Cells (PBMCs and splenocytes) were diluted to a final concentration of 2 million cells per mL. One hundred *μ*L of the cell suspension was added per well in the polyvinyldifluoride (PVDF) membrane 96-well microtiter ELISpot plate.

### 2.6. IFN-*γ* ELISpot

IFN-*γ* ELISpot was performed as described by the manufacturer (Mabtech AB, Nacka, Sweden). Briefly, following anti-IFN-*γ* antibody coating, plates were washed and blocked with RPMI complete medium (RPMI medium containing 1% PEST, 2 mM L-glutamine, 1% HEPES, and 10% FCS). Per well, 200,000 mononuclear cells were added and were stimulated over night with 1 *μ*g of antigen. Detection of spots was done using alkaline-phosphate conjugated antibodies. Substrate was added, and color reaction proceeded for 10 minutes at room temperature and was stopped by extensive wash in tap water. Plates were let to dry and read using the AID ELISpot reader system. Cutoff of responding animals was set to >50 spot-forming cells (SFC)/million cells (PBMCs or splenocytes), and was calculated by subtracting the background response to the HLA-A2 restricted control peptide SLYNTVATL from HIV-1 p17 Gag.

### 2.7. GM-1 Binding Property of the Fusion Proteins

A carboxymethyldextran (CM5; Biacore, Uppsala, Sweden) sensor chip was docked to the BIACORE2000 machine (Biacore, Uppsala, Sweden) and flowed with HBS-EP buffer (10 mM HEPES pH 7.4, 0.15 M NaCl, 3 mM EDTA, and 0.005% Surfactant P20). GM-1 was immobilized on the chip by direct injection, followed by blocking in 1% BSA in HBS-N (10 mM HEPES pH 7.4, 0.15 M NaCl). The samples (rCTB standards, controls, and rCTB-HIV_fusion protein_) were automatically injected to the chip. The amount of bound rCTB was measured as resonance units (RU) and translated into rCTB concentration through the standard curve constructed from known concentrations of rCTB standard (ranging from 2.37 to 0.0925 *μ*g/mL rCTB). GM-1 binding capacities of the fusion proteins were calculated as percent binding in relation to rCTB standard, using the formula

(1) $$\begin{array}{c} \left(\frac{\text{bound fusion protein}}{\text{maximal bound rCTB protein}}\right)\times 100. \end{array}$$

### 2.8. Statistics

Statistical comparisons between groups were performed using the nonparametric Kruskal-Wallis test to detect differences between groups for the ELISpot results. At significant differences, individual groups were compared using the Mann-Whitney *U* test. A two-tailed analysis was performed with the criteria of a significant level of 95%. All calculations were done using the GraphPad Prism v4.02 software (GraphPad Software Inc.). Correlation comparisons were performed with Spearman rank test by correlating binding capacities to GM-1 for the fusion proteins with the individual ELISpot result per mouse using Statistica 8.0 software (StatSoft Inc.). 

## 3. Results and Discussion

**Figure 1:**
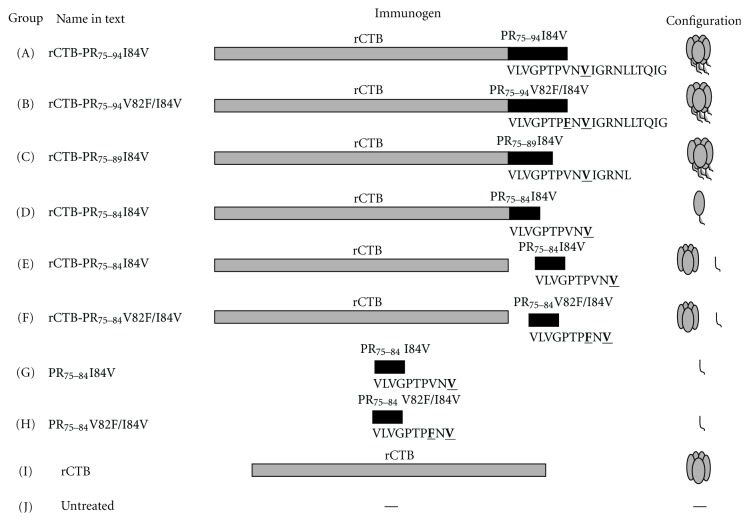
(a)–(i) Schematic picture of the immunogens.

The importance of cytotoxic T cells in HIV infection as well as in SIV/SHIV infection in nonhuman primates is well established [19–24]. Additionally, the potency of CTB as a carrier of protein or peptide for the induction of a humoral immune response has been previously proven [10–12]. However, the capacity of CTB as a carrier for small peptides [9] has not been fully explored. In the present study, we linked an HIV-1 protease epitope to CTB (schematically viewed in Figure 1), which affected the molecular structure of CTB, and tested the immunogenic potency of the resulting fusion protein. The HIV protease peptide was chosen, since it has clinical potential to prevent resistance to certain drug treatments [7, 8].

**Figure 2:**
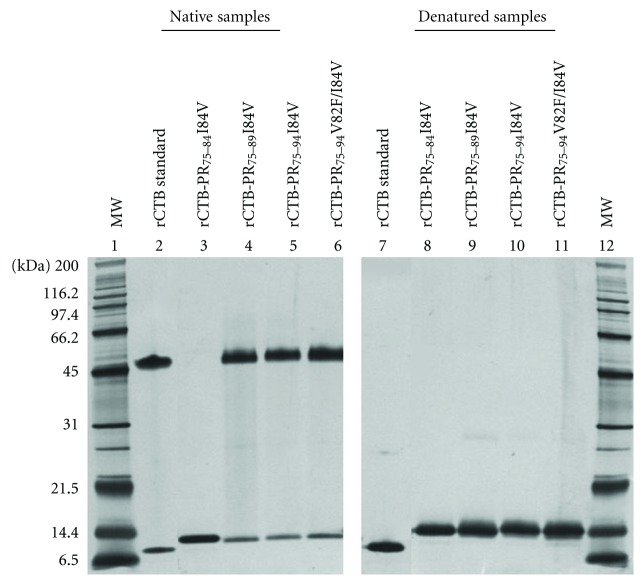
Silver staining of rCTB-protease fusion proteins. Purified protein was run on SDS-PAGE gels and silver stained. Lanes 1–6: native samples. Lanes 7–12: denatured samples. Lanes 1 and 12: see Blue 2 molecular weight standard, Lanes 2 and 7: rCTB standard, Lanes 3 and 8: rCTB-PR_75–84_I84V, Lanes 4 and 9: rCTB-PR_75–89_I84V, Lanes 5 and 10: rCTB-PR_75–94_I84V, and Lanes 6 and 11: rCTB-PR_75–94_V82F/I84V.

**Figure 3:**
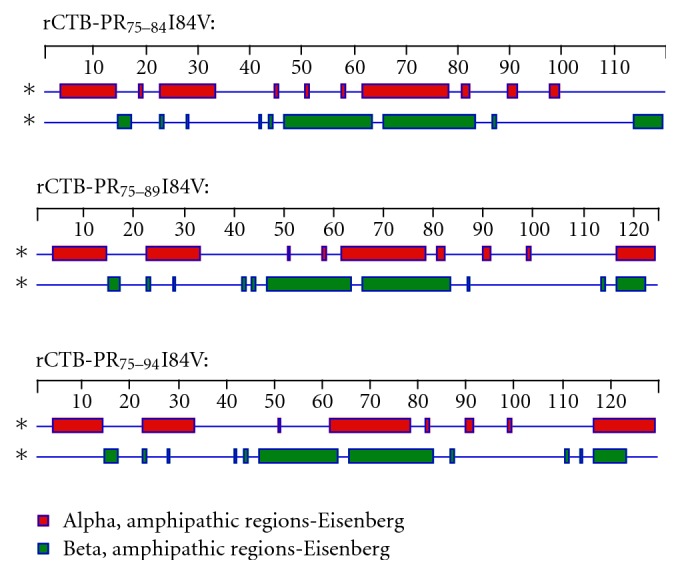
Theoretical prediction of the secondary structure characteristics of the fusion proteins based on the Eisenberg method.

Under native conditions, we found that rCTB-PR_75–89_I84V, rCTB-PR_75–94_I84V, and rCTB-PR_75–94_V82F/I84V fusion proteins mainly formed pentamers (Figure 2, lanes 4, 5, and 6, resp.), while rCTB-PR_75–84_I84V almost exclusively remained in a monomeric configuration (Figure 2, lane 3). The molecular weights of the expressed fusion proteins agreed with the theoretical weight of approximately 13.3 kDa (varying from 12.7–13.8 kDa) under denatured conditions, and molecular weights of approximately 66.5 kDa (varying from 63.5 to 69 kDa) were found for the pentameric proteins under native conditions (Figure 2, lanes 4, 5, and 6). Initial experiments with the monomeric fusion protein showed low immunogenicity. The comparison in the natural structure of that fusion protein in relation to the fusion protein used previously [9] made us construct fusion proteins having a longer epitope attached to the CTB moiety but that retained the natural ability to form pentamers. The individual sequences of the fusion proteins analyzed in the Protean program revealed for all of them that a theoretical alpha amphipathic region scored for the C-terminal part of the protein in at least one algorithm correlated with ability to form pentamers, whereas the original epitope without additional amino acids did not form pentamers and did not score an alpha amphipathic region, Figure 3. To investigate the potency of these new fusion proteins to induce and augment a cellular immune response to the conjugated epitope, HLA-A0201 transgenic C57BL/6 mice were immunized (Table 1), and the epitope-specific immune responses were analyzed.

**Figure 4:**
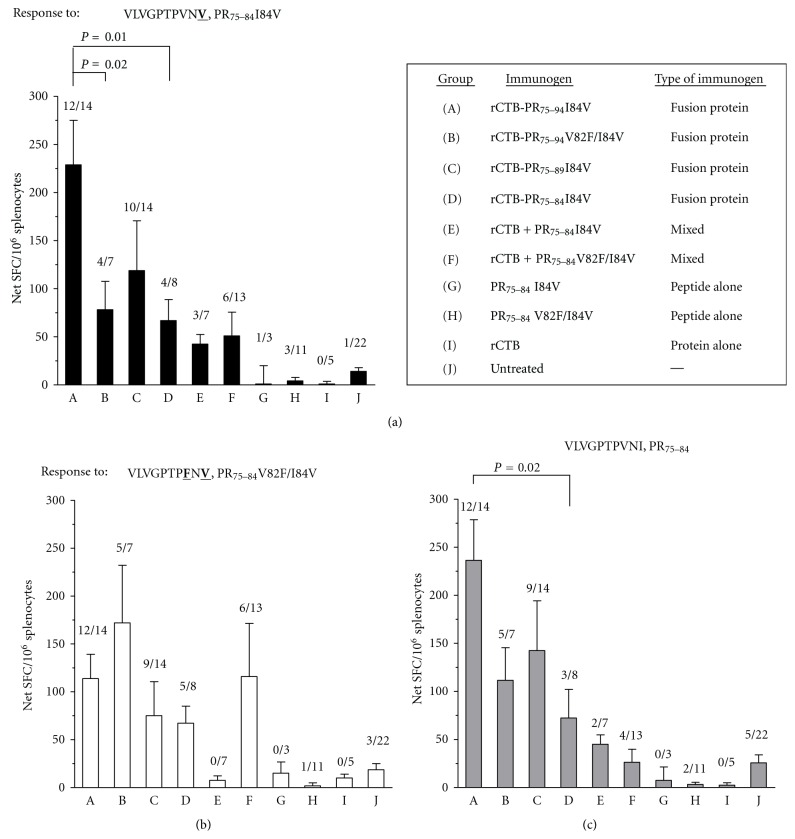
Specific immune responses to HIV PR_75–84_ peptide and variants harbouring drug-resistance mutations. Splenocytes were purified two weeks following the last immunization. The cells were stimulated over night with the (a) singly mutated (I84V), (b) doubly mutated (V82F/I84V), or (c) the wild-type peptide variants of PR_75–84_. Bars are showing the median response of the different groups. Specific responses were calculated by subtracting background responses to a control peptide from PR-specific immune responses. Proportion of responding animals (>50 spot forming cells/10^6^ splenocytes) are indicated above each individual bar.

Immunizing with the pentameric rCTB-PR_74–94_I84V induced significantly higher responses (*P* = 0.01) than the monomeric rCTB-PR_75–84_I84Vor the mixture of rCTB and peptide (Figures 4(a)–4(c), group/s A versus E–H). Immunization with the peptide alone did not induce detectable cellular responses to either epitope (Figures 4(a)–4(c), groups G and H). Interestingly, when comparing the biological property of the CTB moiety of the fusion proteins by measuring binding to GM-1 receptor (Table 2), we observed that the ability to interact with GM-1 correlated with immunogenicity (Figures 4(a)–4(c), groups A–C). A positive correlation was seen between binding to GM-1 and induced immune response against the PR_75–84_I84V, and the wild type PR_75–84_ epitopes (*r* = 0.47, *P* = 0.001, and *r* = 0.4, *P* = 0.01, resp.). No correlation between binding capacity and induced immune response could be observed against the doubly mutated PR_75–84_V82F/I84V epitope.

Taken together, our results imply that the capacity of CTB fusion proteins to be in a pentameric configuration, which is required for GM-1 binding, is crucial in order to induce cellular immune response against the conjugated epitope. It has been shown that conjugation of antigen to CTB enhances the transport of the antigen over mucosal surfaces and an increased delivery of the antigen to macrophages and dendritic cells [25, 26]. Moreover, a retrograde transport of the toxin-receptor complex via the Golgi compartment to the endoplasmic reticulum (ER) follows upon binding of CTB to GM-1 [27–29]. Such targeted ER transport will potentially enhance the chance of peptide/antigen loading onto HLA class I molecules. This possibly explains why the conjugation of an epitope to pentameric CTB enhances the immunogenicity of the antigen, and why immunization with a fusion protein unable to bind GM-1 are less effective in stimulating a cellular immune response [11, 30]. Another mechanism that may play a role in the enhancement of the immune response following administration of the CTB-fusion proteins is the induction of an inflammatory response by CTB [31]. This theory is supported by our findings that the monomeric variant of the fusion protein and the mixture of rCTB and free peptide induced similar magnitudes of immune responses against the protease-derived peptides, whereas injecting the free peptide alone did not stimulate an immune response (Figure 4, groups D–H). 

To investigate the capacity to induce an epitope-specific memory response following immunization with the fusion proteins, we introduced a four-month interruption in the immunization protocol between a third and fourth immunization. The response was measured following three immunizations with rCTB-PR_75–94_I84V(a response of 223 SFC/10^6^ PBMC, data not shown). During the four-month interruption of the immunization protocol, the level of the response was reduced to 50% of the original level. However, the late fourth immunization at week 28 resulted in a rapid anamnestic response (578 SFC/10^6^ PBMC), indicating that an immunological memory was induced by the initial three immunizations. 

Immunization with the rCTB-PR fusion proteins did induce immune responses against not only the epitope variant found in the proteins but also against similar PR_75–84_ epitope variants (PR_75–84_ wild type and PR_75–84_V82F/I84V; Figures 4(b)-4(c)). The magnitude of the response was found to be equal against the epitope in the fusion proteins (I84V) and the wild-type epitope (Figures 4(a) and 4(c)), whereas a lower response was in general measured against the double mutant epitope (V82F/I84V, Figure 4(b)). The latter was still true even when we introduced the additional V82F mutation to the protease epitope of rCTB-PR_75–94_V82F/I84V. A possible explanation for this may be that the additional mutation reduced the binding capacity of the fusion protein to GM-1 from 100% to 74%, Table 2. Moreover, knowledge that the T-cell receptor (TCR) recognition is associated with the central amino acids of the epitope presented by the human leukocyte antigen of class I [32] suggests that the minor difference between the wild type (PR_75–84_) and the singly mutated epitope (PR_75–84_I84V), having the mutation at the edge of the epitope should not directly affect TCR recognition of those epitopes. Therefore, it is possible that the same T-cell clone could recognize both the PR_75–84_, and PR_75–84_I84V epitopes. Such an explanation supports our finding that we could measure equal magnitudes of cellular immune responses against these two epitope variants after immunization with fusion proteins expressing the PR_75–84_I84V epitope. However, the additional mutation (V82F) in PR_75–84_V82F/I84V, located more to the central part of the epitope, possibly negatively affected the interaction with the TCR.

## 4. Conclusion

When the CTB molecule was linked to peptides extended by five or ten amino acids downstream, the CTB-peptide complex readily formed pentamers. Even though the added amino acids buried the epitope of interest within a longer (app. 20 amino acids long) stretch of amino acids, it still was possible to elicit an immune response to the epitope of interest. Thus, we show that the key issue of using CTB as a carrier molecule for augmenting the cellular response to an epitope is to retain the natural pentameric structure of CTB. This structure seems to be more affected by the amino acid composition of the epitope of interest than of the length of the epitope attached. It was possible to induce cellular immune responses against the short HIV-1 protease epitope within a longer stretch of amino acids as long as the CTB molecule allowed forming pentamers. This knowledge is important since it provides a tool to evaluate the potential of a fusion protein including CTB as a carrier. Consistently throughout the study, the highest epitope-specific immune response was observed following immunization with rCTB-PR_75–94_I84V. This fusion protein contained a 20-mer region of the HIV protease, and the CTB moiety retained the ability to form functional pentamers. The fusion protein that contained a 15 amino acid long epitope of HIV protease also induced a good but lower response. These two fusion proteins were found to bind with different affinity to GM-1 and the binding correlated with immunogenicity. Taken together, our results show that peptides from HIV fused to CTB in a manner that allows pentamerization of the protein augments the cellular immune response to the epitope. This concept may be used to induce high and durable cellular immune responses against epitope antigens.

## Figures and Tables

**Table 1:** Summary of rCTB-protease_epitope_ fusion protein immunizations.

Question	Immunized with	Amino acid sequence of linked epitope	Conclusion
Can the immunogenicity of an epitope be enhanced by linking it to recombinant Cholera toxin *β*-subunit (rCTB)?	rCTB-PR_75−89_I84V	rCTB-VLVGPTPVN**V**IGRNL	Linking the HIV derived epitope, PR_75−89/94_, to rCTB efficiently enhanced the immune response to the epitope.
	rCTB-PR_75−94_I84V	rCTB-VLVGPTPVN**V**IGRNLLTQIG	
	rCTB + PR_75−84_V82F/I84V	rCTB + VLVGPTP**F**N**V**	
	PR_75−84_V82F/I84V	VLVGPTP**F**N**V**	
	rCTB	rCTB	
	Untreated	—	

Do we need the pentameric form of the chimeric protein for enhancement of immune response?	rCTB-PR_75−89_I84V	rCTB-VLVGPTPVN**V**IGRNL	Pentamerization, permitting GM-1 binding, of the fusion protein correlated with enhanced immune responses to the attached HIV epitope.
	rCTB-PR_75−94_I84V	rCTB-VLVGPTPVN**V**IGRNLLTQIG	
	rCTB-PR_75−84_I84V (monomeric form)	rCTB-VLVGPTPVN**V**	
	rCTB + PR_75−84_I84V	rCTB + VLVGPTPVN**V**	
	PR_75−84_I84V	VLVGPTPVNV	
	Untreated	—	

Can we broaden the immune response by using a variant of the PR_75−84_ containing two mutated amino acid residues?	rCTB-PR_75−94_V82F/I84V	rCTB-VLVGPTP**F**N**V**IGRNLLTQIG	Addition of the extra mutation reduced the binding property of the fusion protein to GM-1, and resulted in a reduced epitope-specific immune response.
	rCTB + PR_75−84_V82F/I84V	rCTB + VLVGPTPFN**V**	
	PR_75−84_V82F/I84V	VLVGPTPFN**V**	
	rCTB	rCTB	
	Untreated	—	

**Table 2:** Receptor binding property of the CTB^a^ moiety of fusion proteins relative to the binding of unmodified CTB.

Group	Fusion protein	Maximal binding	Actual binding	Percent binding^b^ (%)
A	rCTB-PR_75−94_I84V	0.79 mg/mL	0.79 mg/mL	100
B	rCTB-PR_75−94_V82F/I84V	0.86 mg/mL	0.64 mg/mL	74
C	rCTB-PR_75−89_I84V	0.81 mg/mL	0.75 mg/mL	93
D	rCTB-PR_75−84_I84V	0.80 mg/mL	0 mg/mL	0

^a^CTB: Cholera toxin B subunit.

^b^(bound fusion protein/maximal bound rCTB protein) ∗ 100%.
